# Two new functions in the WormBase Enrichment Suite

**DOI:** 10.17912/W25Q2N

**Published:** 2018-03-27

**Authors:** David Angeles-Albores, Raymond Y.N. Lee, Juancarlos Chan, Paul W. Sternberg

**Affiliations:** 1 Division of Biology and Biological Engineering, Caltech, Pasadena, CA, 91125, USA

**Figure 1.**
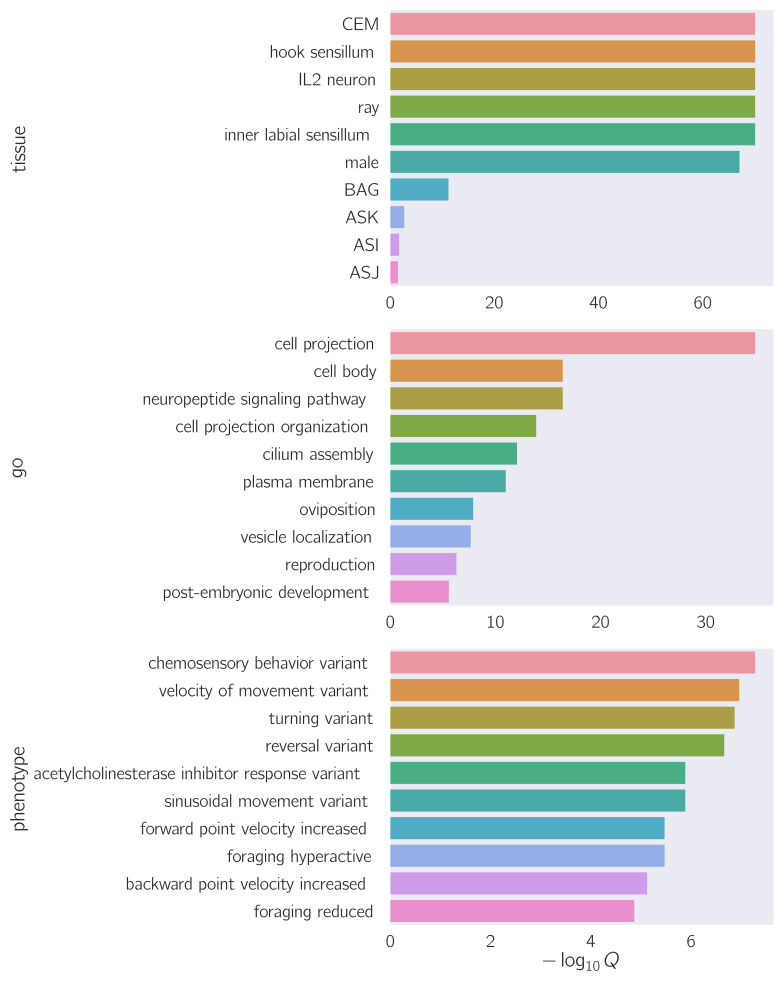
Enrichment results for Tissue, Gene and Phenotype ontologies for genes overexpressed in a ciliary transcriptome (Wang *et al.* 2015).

## Description

**​**Genome-wide experiments routinely generate large amounts of data that can be hard to interpret biologically. A common approach to interpreting these results is to employ enrichment analyses of controlled languages, known as ontologies, that describe various biological parameters such as gene molecular or biological function. In *C. elegans*, three distinct ontologies, the Gene Ontology (GO), Anatomy Ontology (AO), and the Worm Phenotype Ontology (WPO) are used to annotate gene function, expression and phenotype, respectively (Ashburner *et al*. 2000; Lee and Sternberg, 2003; Schindelman *et al*. 2011).

Previously, we developed software to test datasets for enrichment of anatomical terms, called the Tissue Enrichment Analysis (TEA) tool (Angeles-Albores and Sternberg, 2016). Using the same hypergeometric statistical method, we extend enrichment testing to include WPO and GO, offering a unified approach to enrichment testing in *C. elegans*. The WormBase Enrichment Suite can be accessed via a user-friendly interface at http://www.wormbase.org/tools/enrichment/tea/tea.cgi.

To validate the tools, we analyzed a previously published extracellular vesicle (EV)-releasing neuron (EVN) signature gene set derived from dissociated ciliated EV neurons (Wang *et al*. 2015) using WormBase Enrichment Suite based on the WS262 WormBase release. TEA correctly identified the CEM, hook sensillum and IL2 neuron as enriched tissues. The top phenotype associated with the EVN signature was chemosensory behavior. Gene Ontology enrichment analysis showed that cell projection and cell body were the most enriched cellular components in this gene set, followed by the biological processes neuropeptide signaling pathway and vesicle localization further down. The tutorial script used to generate the figure above can be viewed at:https://github.com/dangeles/TissueEnrichmentAnalysis/blob/master/tutorial/Tutorial.ipynb

The addition of Gene Enrichment Analysis (GEA) and Phenotype Enrichment Analysis (PEA) to WormBase marks an important step towards a unified set of analyses that can help researchers to understand genomic datasets. These enrichment analyses will allow the community to fully benefit from the data curation ongoing at WormBase.

## Methods

Using the methods described in Angeles-Albores *et al*, we generated ontology dictionaries using the Anatomy, Phenotype and Gene Ontology annotations for *C. elegans*. The dictionary similarity parameter was set to 95% for all ontologies. The annotation per term minimum was set to 33 annotations for the AO, a 50 annotations for the WPO, and 33 annotations for GO. Terms within the dictionary are tested using a hypergeometric probability test and corrected using the Benjamini-Hochberg step-up algorithm. In WS262, there are 1320 anatomy terms, 1117 phenotypes, and 3025 GO terms that have at least 11 genes annotated to them. The dictionaries are freely accessible using the Python version of the Suite, which can be installed using the pip tool for Python libraries:

pip install tissue_enrichment_analysis

The dictionary can then be automatically downloaded by importing the enrichment analysis library in a Python script by writing:

import tissue_enrichment_analysis as ea

The dictionaries can then be downloaded by typing:

ea.fetch_dictionary(dict)

into Python, where `dict ` is the string `tissue`, `phenotype` or `go` to specify which dictionary to download. If the function does not receive an argument, the dictionary corresponding to the AO is downloaded by default. See the tutorial above for an example implementation.
